# Usefulness of Dual-Energy Computed Tomography Imaging in the Differential Diagnosis of Sellar Meningiomas and Pituitary Adenomas: Preliminary Report

**DOI:** 10.1371/journal.pone.0090658

**Published:** 2014-03-03

**Authors:** Lian-Ming Wu, Yu-Lai Li, Yu-Hua Yin, Guo-Qiang Hou, Rong Zhu, Xiao-Lan Hua, Jian-Rong Xu, Zeng-Ai Chen

**Affiliations:** 1 Department of Radiology, Renji Hospital, Shanghai Jiao Tong University School of Medicine, Shanghai, China; 2 Department of Neurosurgery, Renji Hospital, Shanghai Jiao Tong University School of Medicine, Shanghai, China; Northwestern University Feinberg School of Medicine, United States of America

## Abstract

**Objective:**

To quantitatively assess the imaging characteristics of sellar lesion in dual-energy computed tomography (CT) imaging for differentiation of sellar meningiomas and pituitary adenomas during the arterial phase (AP) and venous phase (VP).

**Materials and Methods:**

51 patients with sellar/parasellar tumors (33 macroadenomas and 18 meningiomas) were examined with CT spectral imaging during the AP and the VP. Iodine concentrations were derived from iodine-based material-decomposition CT images and normalized to the iodine concentration in the aorta. The difference in Normalized iodine concentrations (NICs), HU curve slope (λHU), and mean CT values of lesions between the AP and VP were calculated. The two-sample t test was performed to compare quantitative parameters between sellar meningiomas and pituitary adenomas.

**Results:**

NICs, λHU, and mean CT values in patients with sellar meningiomas differed significantly from those in patients with pituitary adenomas: Mean NICs were 43.52 mg/mL±1.35 versus 9.23 mg/mL ±2.44, respectively, during the AP and 52.13 mg/mL ±1.04 versus 24.37 mg/mL ±2.23 respectively, during the VP. λHU were −3.03±3.42 versus −0.53±0.23, respectively, during the AP and −2.96±0.41 versus −0.47±0.25, respectively, during the VP. Mean CT values were 193.63±2.08 versus 63.98±2.85, respectively, during the AP and 203.98±0.18 versus 77.66±0.91, respectively, during the VP. The combination of NIC and Mean CT value during VP had highest sensitivity (90.9%) and specificity (100%) among all phases.

**Conclusion:**

Quantitative dual-energy CT imaging has promising potential for diagnostic differentiation of sellar meningiomas and pituitary adenomas.

## Introduction

Sellar/parasellar tumors constitute 10–15% of all primary intracranial neoplasms and are the most common causes of pituitary dysfunction and field of view disturbance [1], [2]. Therefore, early correct diagnosis and therapy of patients with sellar/parasellar tumors are of high importance in clinical practice. The most common pituitary tumors are adenomas, which on MRI can present various enhancement patterns and other imaging features. About 18% of macroadenomas contain cystic components, about 20% show foci of hemorrhage, which are usually clinically asymptomatic and diagnosed incidentally in MR imaging [1], [2]. Calcifications in pituitary adenomas are reported in 0.2–8% of cases [3]. Therefore pituitary adenomas may be mimicked by other tumors located in the sellar region, such as meningiomas, craniopharyn-giomas, Rathke cleft cysts, metastases, gliomas, abscesses, as well as uncommon types of sellar/parasellar tumors like hemangioblas-toma. Although MRI is the imaging study of choice for evaluation of sellar lesion or in the context of known or highly suspected pathology, the MR appearance of different sellar/parasellar lesions may be very similar, which often leads to misdiagnosis. The proper choice of surgical approach requires the correct preoperative diagnosis of sellar/parasellar tumors [1], [4]. Computed tomography (CT) is more widely available, is better suited for rapid screening and can be used when patients have MRI exclusions (such as pacemakers). As such, many sellar lesion are first encountered on CT scans obtained for different reasons. Furthermore, the special anatomical location of the pituitary in the sellar region with adjacent bones and air structures (sinuses) may lead to some problems with MRI acquisition, which is very prone to artifacts. Smaller intrasellar and tumors with only a small amount of suprasellar extension will not be able to be evaluated due to the susceptibility artifacts at the skull base. Consequently, CT has a place in the diagnosis of sellar lesion because it is superior in demonstrating the effects of this neoplasm on adjacent bone.

Recently, a novel CT scan mode was introduced, which used dual-energy x-rays produced by the rapid switching of high- and low-tube voltages within a rotation, which allowed precisely registered data sets for creating accurate material decomposition images (eg, water- and iodine-based material decomposition images) and pseudomonochromatic images, with energies ranging from 40 to 140 keV. The material decomposition images can be used to estimate quantitatively the water and iodine content [5] (or concentration at mg/mL) in lesions and normal tissues.

The aim of this work was to assess the feasibility and diagnostic value of detecting and characterizing sellar meningiomas and pituitary adenomas through quantitative dual-energy CT imaging.

## Materials and Methods

### 2.1. Patients

This study was approved by our institutional review board (Renji Hospital, Shanghai Jiao Tong University School of Medicine, Shanghai) and written informed consent was obtained from all patients. From June 2011 to March 2013, 80 patients known or suspected to have Sellar/parasellar tumors underwent multiple-phase CT scanning in the spectral imaging mode, which was performed by using a Discovery CT750 HD CT scanner (GE Healthcare).Patients who had reliable proof of sellar meningiomas and pituitary adenomas were included. Twenty-nine (36%) of the 80 patients were excluded from the study because (a) there was inadequate confirmation of histologic findings (n = 16) (b)Three patients with adenomas were excluded from further analysis because of intratumoral bleeding (n = 3) (c) Other tumors(craniopharyn-giomas,n = 5; squamous-papillary type,n = 2; intrasellar hemangioblastoma,n = 1;intrasellar prostate cancer metastasis,n = 1; suprasellar glioma,n = 1).A total of 51 patients with sellar/parasellar tumors (33 macroadenomas and 18 meningiomas) (age range,38–69 years; mean age, 53 years) were included in our study: 29 were men (age range, 38–61 years; mean age,46 years), and 22 were women (age range,43–69 years; mean age, 57 years). The men were significantly younger than the women (P<0 .05). A total of 51 lesions (mean diameter, 2.3 cm; diameter range, 0.8–2.7 cm) were included.

### 2.2. CT Examinations

Non-enhanced and two-phase contrast material–enhanced CT examinations were performed by using the GE Discovery CT750 HD scanner. All patients were scanned craniocaudally while in the supine position. After scout CT scanning, non-enhanced helical scanning was performed in the conventional helical mode at a tube voltage of 120 kVp. Patients were then injected with iodinated nonionic contrast material (Iopamidol, 370 mg/mL;Shanghai Bracco Sine Pharmaceutical, China) with antecubital venous access at a rate of 4 mL/s and 1.5 mL/kg body weight during the AP and VP. The scanning delay for late AP imaging was determined by using automated scantriggering software (SmartPrep; GE Healthcare). AP scanning automatically began 12 seconds after the trigger attenuation thresh old (100 HU) was reached at the level of the internal carotid. At a delay of 30 seconds after AP scanning, VP scanning began. AP and VP scanning was performed in the spectral imaging mode with fast tube voltage switching between 80 and 140 kVp on adjacent views during a single rotation. Other scanning parameters were as follows: collimation thickness of 0.625 mm, tube current of 600 mA, rotation speed of 0.6 second, helical pitch of 0.983, and CT dose index volume of 21.8 mGy (comparable to 21.5-mGy dose administered for conventional contrast-enhanced scanning in a normal -size patient [with body mass index within normal range] at our institution). The CT images were reconstructed by using projection-based material-decomposition software and a standard reconstruction kernel. The adaptive statistical iterative reconstruction algorithm was applied to suppress image noise on the decomposition images. Three types of images were reconstructed from the single spectral CT acquisition for analysis: conventional polychromatic images obtained at 140 kVp, water- and iodine-based material decomposition images, and monochromatic images obtained at energies ranging from 40 to 140 keV.

### 2.3. Quantitative Analysis

For homogenous lesions, regions of interest (ROIs) could be drawn as big as possible to cover most of the lesion region. For inhomogeneous lesions, ROIs were placed on the regions corresponding to solid components of lesion, and those regions with evident features of cystic or necrotic change were avoided. Two residents blinded to the diagnosis drew the ROIs independently, and in questionable cases, an attending radiologist was consulted to reach consensus. To ensure consistency, all measurements were performed three times at different image levels, and average values were calculated. For each tumor, circular or elliptical regions of interest were drawn to encompass as much of the high-enhancing portion of the lesion as possible. For all measurements, the size, shape, and position of the regions of interest were kept consistent between the two phases by applying the copy-and-paste function. Quantitative parameters during the AP and VP included the following: (a) To minimize variations in patients, scanning times, and iodine concentrations, the iodine concentration in the lesion was normalized to the iodine concentration in the artery to derive a normalized iodine concentration (NIC); (b) slope of HU curves (λHU) (the plot of material attenuation against x-ray photon energy) corresponding to tumor calculated as the CT attenuation difference at 2 energy levels (40 and 100 keV) divided by the energy difference (60 keV) from the HU curve: λHU = (HU40 kev-HU100 kev)/60; (c) mean CT values of the tumor.

### 2.4. Statistical Analyses

All the data was normal distribution. The two-sample t test was performed to compare the parameters NIC, λHU, and mean CT value between sellar meningiomas and pituitary adenomas during AP and VP,with P<0 .05 indicating significance. Receiver operating characteristic curves [6] were generated to help establish the threshold values of these parameters required for significant differentiation of sellar meningiomas from pituitary adenomas. True-positive cases were defined as those in which sellar meningiomas was correctly diagnosed. False-positive cases were defined as those in which pituitary adenomas was misdiagnosed as sellar meningiomas. The diagnostic capability was determined by calculating the area under each reader-specific receiver operating characteristic curve. A receiver operating characteristic (ROC) is a graphical plot which illustrates the performance of a binary classifier system as its discrimination threshold is varied. It is created by plotting the fraction of true positives out of the total actual positives vs. the fraction of false positives out of the total actual negatives at various threshold settings. The best sensitivity and specificity were achieved by using the optimal thresholds. Optimal sensitivity and specificity were defined as the maximal sensitivity and maximal specificity values. The statistical analyses were performed by using statistical software (SPSS for Windows, version 18.0; SPSS Inc.,Chicago, IL).The null hypothesis tested was that the area under the receiver operating characteristic curve was 0.5; the alternative was that this area was greater than 0.5.

## Results

**Figure 1:**
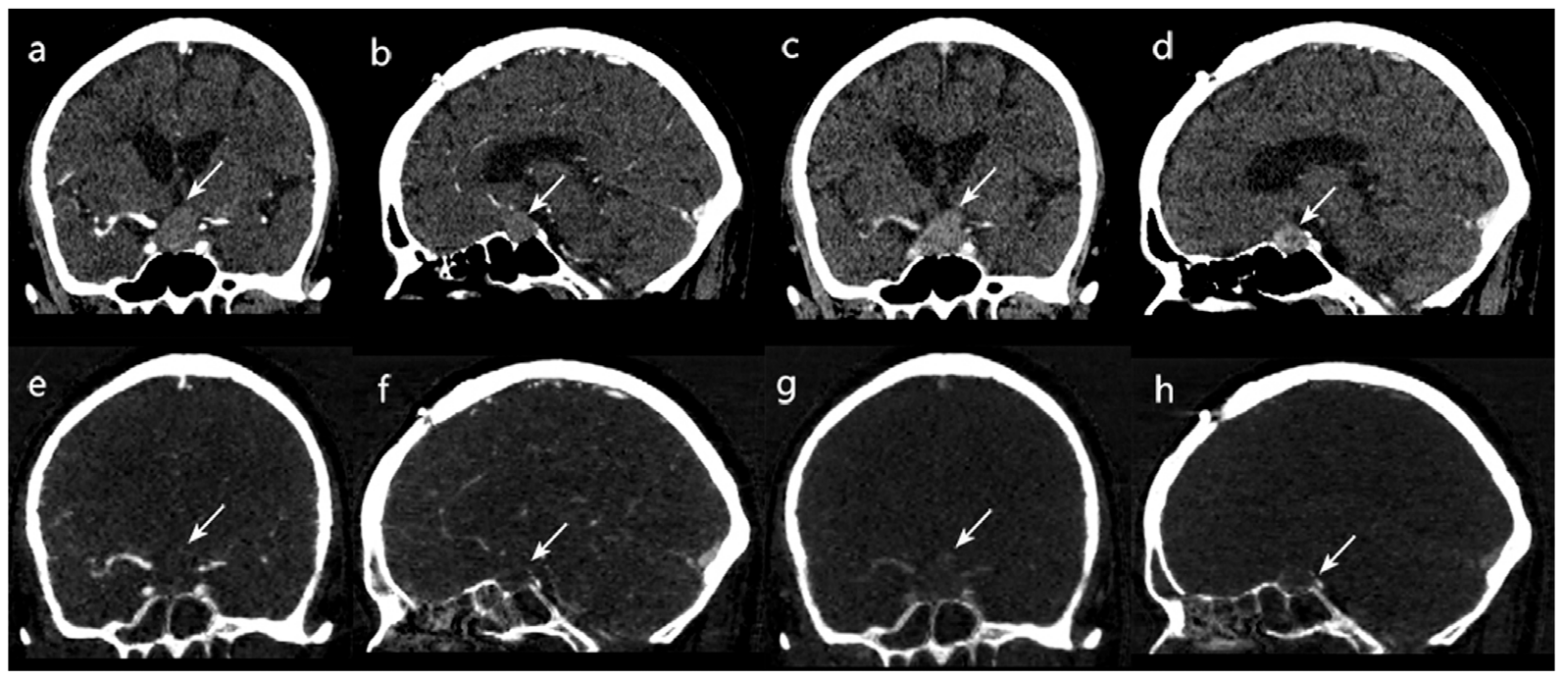
A 65-year-old man with pathologically confirmed pituitary adenoma. Monochromatic CT images obtained at 75-keV energy level (a) Coronal arterial phase, (b) Sagittal arterial phase.(c) Coronal venous phase (d) Sagittal venous phase. iodine-based material decomposition images from single spectral CT acquisition (E) Coronal arterial phase, (F) Sagittal arterial phase.(G) Coronal venous phase (H) Sagittal venous phase.

The results of the calculated NICs, λHUs and CT values as well as the location of the tumour were shown in Table S1. Values for the defined quantitative parameters (NIC, λHU, and mean CT value) measured in the patients with sellar meningiomas and pituitary adenomas are compared in Table 1. There were significant differences in NIC between the patients with sellar meningiomas and the patients with pituitary adenomas during both the AP (mean NIC, 43.52 +1.35 mg/mL vs 9.23 +2.44 mg/mL; P = 0.006) and the VP (mean NIC, 52.13+1.04 mg/mL vs 24.37+2.23 mg/mL; P<.001). There were significant differences in λHU between the patients with sellar meningiomas and the patients with pituitary adenomas during both the AP (λHU, −3.03+3.42 vs −0.53+0.23; P<.001) and the VP (λHU, −2.9679+0.41 vs −0.47+0.25; P<.001). The patients with sellar meningiomas had significantly higher mean CT value than did the patients with pituitary adenomas during the AP (mean CT value, 193.63+2.08 vs 63.98+2.85; P<.001) and the VP (mean CT value, 203.98+0.18 vs 77.66+0.91; P<.001). Two example set of images derived from a single spectral CT acquisition in two patient with sellar meningiomas or pituitary adenomas is shown in Figure 1,2.The areas under the reader-specific receiver operating characteristic curves for all parameters for differentiating between sellar meningiomas and pituitary adenomas —especially those for the NIC AP (0.91) and the mean CT value VP (0.94) were greater than 0.90.(Figure3).By using the receiver operating characteristic curves, we determined the parameter threshold values required to optimize both the sensitivity and the specificity for differentiating between sellar meningiomas and pituitary adenomas (Table2). For example, during the AP, a threshold NIC of 11.85 mg/ML would yield sensitivity and specificity of 84.8% and 94.4%.However, during the VP, a threshold NIC of 22.8 mg/mL would increase the sensitivity of 90.9% and decrease specificity of 88.9%, respectively.

**Figure 2:**
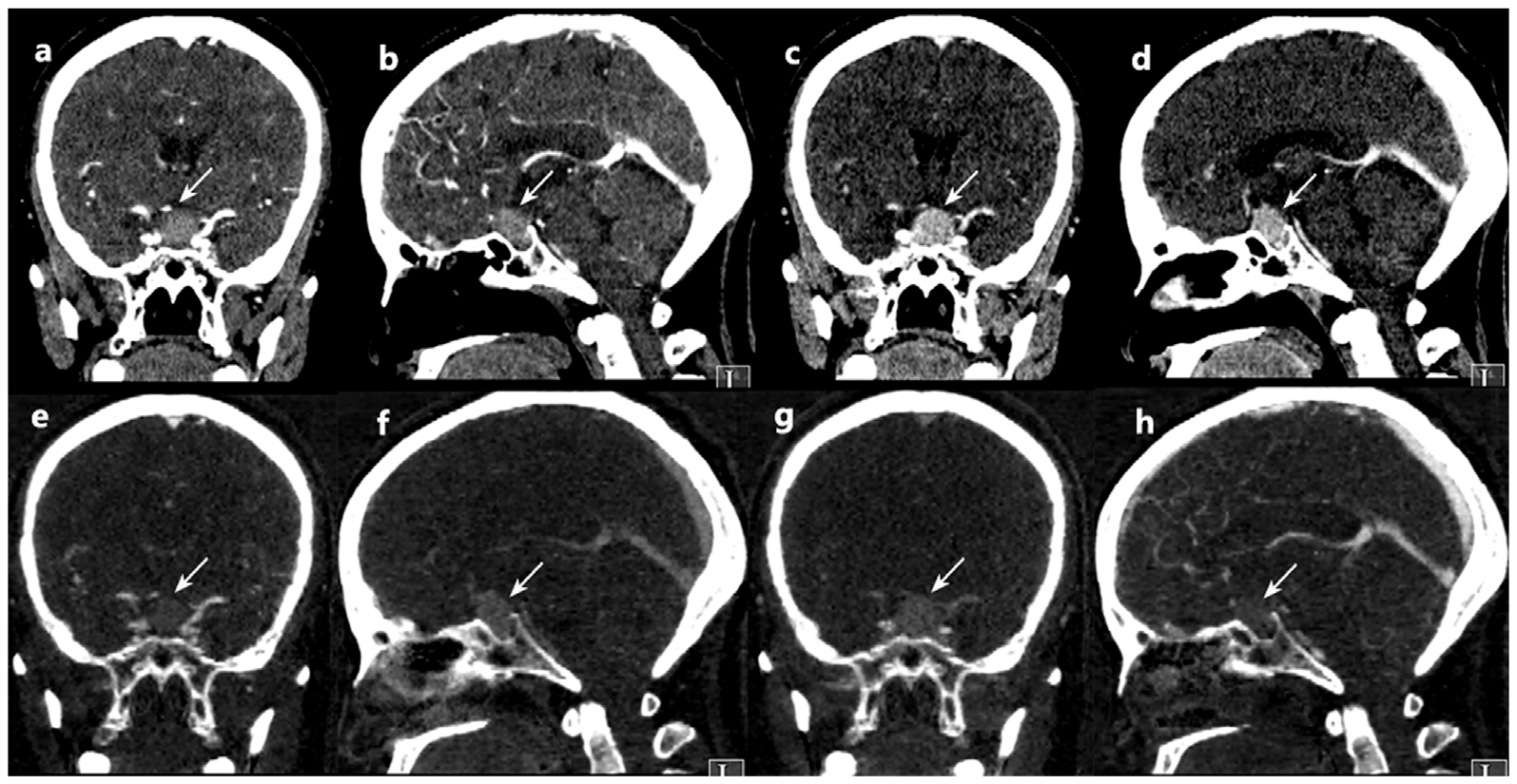
A 46-year-old man with pathologically confirmed sellar meningioma. Monochromatic CT images obtained at 75-keV energy level (a) Coronal arterial phase, (b) Sagittal arterial phase.(c) Coronal venous phase (d) Sagittal venous phase. iodine-based material decomposition images from single spectral CT acquisition (E) Coronal arterial phase, (F) Sagittal arterial phase.(G) Coronal venous phase (H) Sagittal venous phase.

**Figure 3:**
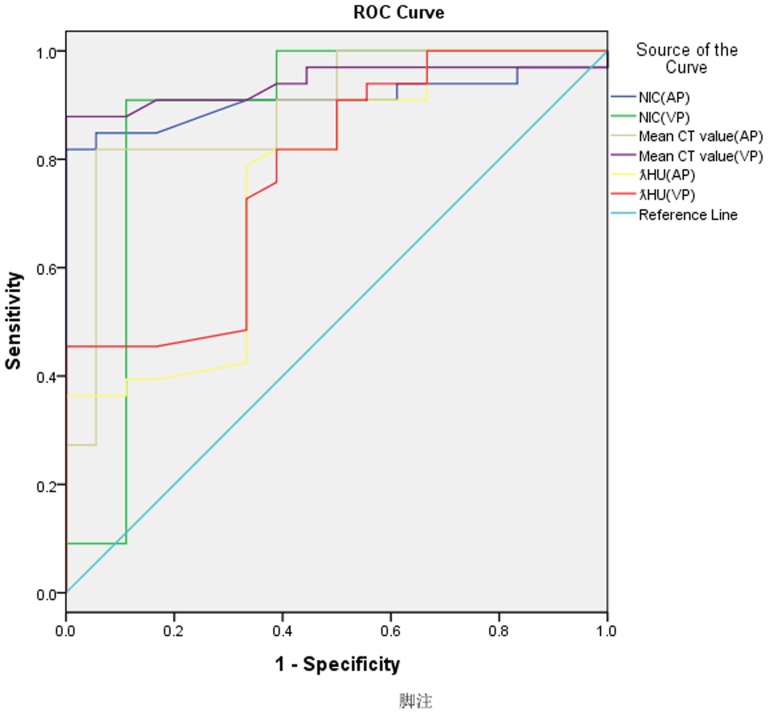
Receiver operating characteristic curves for NIC, λHU and mean CT value in differentiating pituitary adenoma from sellar meningioma during AP and VP.

**Table 1:** Quantitative Assessment of sellar meningiomas and pituitary adenomas at CT Spectral Imaging.

Parameter	Sellar meningiomas(n = 18)	Pituitary adenomas(n = 33)	P
AP			
NIC (mg/mL)	43.52±1.35	9.23±2.44	0.006
HU curve slope	−3.03±3.42	−0.53±0.23	<0.001
Mean CT value	193.63±2.08	63.98±2.85	<0.001
VP			
NIC (mg/mL)	52.13±1.04	24.37±2.23	<0.001
HU curve slope	−2.9679±0.41	−0.47±0.25	<0.001
Mean CT value	203.98±0.18	77.66±0.91	<0.001

**Table 2:** Thresholds, Sensitivities, and Specificities for Distinguishing sellar meningiomas from pituitary adenomas.

Parameter	Threshold Value	Sensitivity	Specificity
NIC during AP	11.85	84.8	94.4
HU curve slope during AP	0.36	81.8	61.1
Mean CT value during AP	82.4	81.8	94.4
NIC during VP	22.8	90.9	88.9
HU curve slope during VP	0.39	72.8	66.7
Mean CT value during VP	86.45	87.9	100

In terms of selected combinations of optimal thresholds, the combination of NIC and Mean CT value during VP (22.8 mg/mL and 86.45, respectively) yielded optimal sensitivities and specificities of 90.9% and 100%, respectively, for differentiating between sellar meningiomas from pituitary adenomas.

## Discussion

Pituitary adenomas comprise over 90% of sellar masses [7]. Meningiomas comprise approximately 18% of all intracranial neoplasms and represent the second most common sellar region lesion behind pituitary adenomas [8]. The vast majority of the CT studies of intracranial tumors have focused on glialneoplasms. Only some articles have reported the usefulness of CT in the differentiation of various extra-axial tumors, especially meningiomas [9]. Furthermore, most of the studies concerning intracranial tumors, draw special attention to qualitative assesment without quantitative parameters.

The averaging attenuation effect of polychromatic x-rays at conventional CT reduces the low-contrast spatial resolution between materials. In addition, beam hardening caused by the preferential absorption can shift the attenuation of a material in the scanning field of view within a patient or between patients. Because of beam-hardening artifacts, attenuation values are sometimes unreliable for verification of enhancing versus nonenhancing small lesions. On the other hand, the use of a monochromatic x-ray beam in CT would eliminate the beam-hardening artifacts and averaging attenuation effects. CT spectral imaging yields monochromatic images that depict how the imaged object would look if the x-ray source produced only single-energy x-ray photons. This would facilitate increasing contrast spatial resolution.

For medical diagnostic imaging, water and iodine are often selected as the basis pair for material-decomposition image presentation because their atomic numbers span the range of atomic numbers for materials generally found in medical imaging and approximate those of soft tissue and iodinated contrast material to result in material-attenuation images that are intuitive to interpret. The iodine concentration in lesions de-rived from the iodine-based material decomposition images is quantitative, as demonstrated in the in Lv's study [10], and thus might be a useful parameter.

To the best of our knowledge, this report is the first study evaluating dual-energy CT imaging of pituitary and parasellar tumors, including the assessment NICs, λHU, and means CT values measurements. According to our study results, Statistical analysis showed NICs, λHU, and mean CT values in patients with sellar meningiomas differed significantly from those in patients with pituitary adenomas. The NICs derived from the iodine-based material decomposition images satisfactorily represented contrast medium uptake in pituitary and parasellar lesions, which in turn depicts characteristics of the blood supply. Using quantitative iodine concentration measurements, the sensitivity and specificity for differentiating sellar meningiomas with pituitary adenomas reached 84.8% and 94.4% during the AP, 90.9%, and 88.9% during the VP, respectively. The uptake or washout of contrast material is represented by variations in iodine concentration during the two continuous phases in our study. According to our study results, the NIC was higher in enhancing sellar meningiomas than in enhancing pituitary adenomas in both AP and VP phase. These results suggest that pseudomonochromatic imaging reconstruction and material decomposition-based quantitative dual-energy CT imaging have promising potential for diagnostic differentiation of sellar meningiomas from pituitary adenomas. However, more investigation is required to determine the optimal iodine, HU curve slope (λHU), and mean CT values threshold for the best differentiation.

There are some limitations of our study due to the small number of sellar/parasellar tumors other than adenomas and meningiomas. Nevertheless, this is the data from a preliminary pilot study that shows the NICs, HU curve slope (λHU), and mean CT values between pituitary adenomas and meningiomas were dramatically different and had clinical relevance. Second, this study was focused on the use of quantitative information generated by using CT spectral imaging. In the future, qualitative assessment of iodine- and water-based images could be added to evaluate their clinical value. Third, because the readers assessed the images in consensus, we do not have data on intra- or interobserver variability.

In conclusion, dual-energy CT imaging provides promising quantitative approach for distinguishing sellar meningiomas with pituitary adenomas. Based on this study, iodine content, slope of HU curve, and mean CT value could be valuable parameters for sellar meningiomas and pituitary adenomas differentiation, which warrants further preoperative-postoperative radiologic-pathologic comparison imaging study.

## Supporting Information

Table S1 **The results of the calculated NICs, λHUs and CT values as well as the location of the tumour.**
(DOC)
