# Supplementary material for: Usefulness of Dual-Energy Computed Tomography Imaging in the Differential Diagnosis of Sellar Meningiomas and Pituitary Adenomas: Preliminary Report
Source: PLoS One. 2014 Mar 3;9(3):e90658. doi: 10.1371/journal.pone.0090658 (PMC3940935; doi:10.1371/journal.pone.0090658)
Supplement: Table S1 — The results of the calculated NICs, λHUs and CT values as well as the location of the tumour. (DOC) [file pone.0090658.s001.doc]

Supporting information:The results of the calculated NICs, λHUs and CT values as well as the location of the tumour.

| Macroadenomas | NICAP | NICVP | HU AP | HU VP | CT value AP | CT value VP | Tumor location |
| --- | --- | --- | --- | --- | --- | --- | --- |
| Patient 1 | 42 | 70 | 1.07 | 1.07 | 134.3 | 221.8 | intrasellar |
| Patient 2 | 40 | 72 | 1.07 | 1.07 | 134.4 | 223.9 | suprasellar |
| Patient 3 | 39 | 72 | 0.93 | 0.93 | 138.3 | 224.8 | intrasellar |
| Patient 4 | 28 | 36 | 0.2 | 0.2 | 95.4 | 120.2 | intrasellar |
| Patient 5 | 25 | 37 | 0.2 | 0.2 | 94.2 | 122.3 | intrasellar |
| Patient 6 | 27 | 36 | 0.2 | 0.2 | 101 | 123 | intrasellar |
| Patient 7 | 27 | 28 | 0.45 | 0.45 | 106.2 | 103.4 | intrasellar |
| Patient 8 | 31 | 30 | 0.5 | 0.5 | 117.8 | 119.6 | suprasellar |
| Patient 9 | 34 | 31 | 0.6 | 0.6 | 135.6 | 124.2 | parasellar |
| Patient 10 | 26 | 34 | 0.4 | 1.3 | 91.3 | 115.9 | intrasellar |
| Patient 11 | 29 | 35 | 0.56 | 1.2 | 100.1 | 117.8 | suprasellar |
| Patient 12 | 21 | 23 | 0.73 | 1.7 | 83.8 | 91.6 | intrasellar |
| Patient 13 | 122 | 125 | 1.5 | 1.5 | 622.3 | 625.5 | suprasellar |
| Patient 14 | 164 | 169 | 1.9 | 1.9 | 825 | 833.7 | intrasellar |
| Patient 15 | 180 | 183 | 2.35 | 2.35 | 892.7 | 901.9 | intrasellar |
| Patient 16 | 70 | 81 | 1.33 | 1.08 | 340.8 | 168.7 | intrasellar |
| Patient 17 | 64 | 84 | 1.26 | 1.54 | 314.7 | 382.7 | intrasellar |
| Patient 18 | 54 | 73 | 1.15 | 1.08 | 272.8 | 368.3 | intrasellar |
| Patient 19 | 24 | 34 | 0.02 | 0.05 | 101.6 | 132 | intrasellar |
| Patient 20 | 21 | 33 | 0.04 | 0.05 | 99.5 | 132 | suprasellar |
| Patient 21 | 22 | 34 | 0.01 | 0.13 | 101.7 | 135 | intrasellar |
| Patient 22 | 70 | 41 | 0.9 | 0.9 | 208 | 160.3 | intrasellar |
| Patient 23 | 56 | 54 | 1.45 | 1.45 | 267.7 | 206.9 | intrasellar |
| Patient 24 | 82 | 68 | 1.6 | 1.6 | 314.6 | 26.3 | intrasellar |
| Patient 25 | 27 | 34 | 0.5 | 0.5 | 101.9 | 122 | suprasellar |
| Patient 26 | 29 | 35 | 0.4 | 0.4 | 106.7 | 126 | intrasellar |
| Patient 27 | 31 | 37 | 0.38 | 0.38 | 113.6 | 131 | intrasellar |
| Patient 28 | 11 | 14.5 | 0.55 | 0.4 | 67.5 | 77 | intrasellar |
| Patient 29 | 12 | 15.3 | 0.54 | 0.35 | 71.2 | 80 | parasellar |
| Patient 30 | 11 | 13.5 | 0.55 | 0.35 | 68 | 75 | intrasellar |
| Patient 31 | 7.4 | 29 | 0.42 | 0.5 | 54.8 | 113.4 | intrasellar |
| Patient 32 | 8.9 | 29 | 0.5 | 0.5 | 56.8 | 113.2 | intrasellar |
| Patient 33 | 1 | 30 | 0.5 | 0.47 | 55.4 | 112 | intrasellar |
| Meningiomas | NICAP | NICVP | HU AP | HU VP | CT value AP | CT value VP | Tumor location |
| Patient 1 | 11 | 21.4 | 0.14 | 0.14 | 46.5 | 75.9 | parasellar |
| Patient 2 | 10 | 21.5 | 0.08 | 0.08 | 47 | 78.7 | parasellar |
| Patient 3 | 11 | 22.6 | 0.11 | 0.11 | 48 | 81.3 | intrasellar |
| Patient 4 | 10.1 | 6.8 | 0.23 | 0.23 | 52.7 | 63 | parasellar |
| Patient 5 | 11.7 | 7.2 | 0.34 | 0.34 | 57.6 | 70 | parasellar |
| Patient 6 | 11.4 | 7.1 | 0.38 | 0.38 | 60 | 72 | parasellar |
| Patient 7 | 8.3 | 8.3 | -0.05 | -0.05 | 76.8 | 77 | intrasellar |
| Patient 8 | 10 | 9.9 | -0.03 | -0.03 | 81 | 81 | parasellar |
| Patient 9 | 9.8 | 9.8 | -0.12 | -0.12 | 80 | 80 | parasellar |
| Patient 10 | 11 | 16 | 0.6 | 0.6 | 78.8 | 78.8 | parasellar |
| Patient 11 | 13.4 | 16 | 0.6 | 0.6 | 78.3 | 78.3 | intrasellar |
| Patient 12 | 9.7 | 12 | 0.6 | 0.6 | 71.8 | 71.8 | suprasellar |
| Patient 13 | 5.4 | 7 | 0.65 | 0.65 | 46.1 | 46.1 | parasellar |
| Patient 14 | 4.3 | 3.7 | 0.75 | 0.75 | 45.5 | 45.4 | parasellar |
| Patient 15 | 5.2 | 6 | 0.75 | 0.75 | 161.6 | 51.2 | intrasellar |
| Patient 16 | 7.8 | 86 | -0.44 | -0.44 | 38 | 45 | parasellar |
| Patient 17 | 8.6 | 86 | -0.94 | -0.94 | 42 | 37 | parasellar |
| Patient 18 | 7.5 | 8.3 | -0.3 | -0.3 | 40 | 42 | parasellar |
